# Membranes for Water, Gas and Ion Separation

**DOI:** 10.3390/membranes11050325

**Published:** 2021-04-29

**Authors:** Seungju Kim

**Affiliations:** Department of Chemical Engineering, The University of Melbourne, Parkville, VIC 3010, Australia; seungju.kim@unimelb.edu.au

In recent years, many industry sectors have recognised the importance of sustainable energy, reducing energy consumption and efficient production. In light of the international recognition and demand for efficient energy consumption, membrane separation technology has been developed as part of the vital elements in those footsteps. Advanced membrane technology enables a green process for capturing carbon dioxide or separating hydrogen for reducing greenhouse gas emissions. In addition, membrane separation has been applied in diverse industrial processes, including chemical, food, pharmaceutical and wastewater treatment. The versatility of this technology cannot be emphasised enough. To date, multifaceted membranes are in the process of development, as are the types of materials of membranes. The more that advanced and sustainable membranes develop, the more that efficient and greener processes will be available to meet the global need to act on climate change. 

This Special Issue titled “Membranes for Water, Gas and Ion Separation” in the journal *Membranes* aims to assess recent developments in sustainable and environmentally-friendly membrane technology in depth. Various topics are discussed, including membrane fabrication, pervaporation, organic solvent nanofiltration, gas separation and water recovery. There are five contributions, namely two research articles and three reviews, in this Special Issue.

Dibrov et al. [1] prepared polysulfone (PSf) with controlled pore size distributions by hypochlorite treatment. As PSf is one of the most widely applied membrane materials, the facile morphology controls without applying chemical agents can be advantageous to obtain preferred membrane properties economically. Moreover, because sodium hypochlorite solution in concentrations up to 400 ppm is typically used for membrane washing, the effect of these treatments on membrane properties should be considered for long-term operation using PSf membranes. First, PSf hollow fibre membranes were prepared by the non-solvent induced phase separation method from the dope solution containing poly(ethylene glycol) and polyvinylpyrrolidone, which is a common agent to increase hydrophilicity and water permeance, to induce the pores for ultrafiltration. The prepared hollow fibre membranes presented an initial water flux of 270 L m^−2^ h^−1^ bar^−1^. After hypochlorite treatment at 50 to 5000 ppm, it increased to 500–1400 L m^−2^ h^−1^ bar^−1^. Water flux reached a maximum by treatment at 50 ppm. The treatment conditions influenced pore sizes and porosity of the membrane surfaces, since hypochlorite degraded polyvinylpyrrolidone and residual PSf from the membrane matrices. 

Yan et al. [2] investigated sulfonated styrene-ethylene/butylene-styrene (S-SEBS) membranes with improved mechanical properties by crosslinking with hyperbranched polyester (H302). S-SEBS is a type of sulfonated aromatic polymer that presents high water transport properties due to hydrophilic functional groups and also presents extreme hydrophobicity due to the polymer backbone structure. The resulting membranes were characterised by various techniques including TIR, DSC, EA, SEM, TEM and SAXS. The microphase separation from these domains formed continuous nanochannels for favourable water diffusion. However, excessive swelling weakened the mechanical properties of highly sulfonated membranes. Their approach to address this was to crosslink by hyperbranched polymers. Due to the highly affinitive nature between sulfonated groups in S-SEBS and hydroxyl groups in H302, crosslinking could occur simply by blending for hydrogen bonding or by solid-state thermal treatment for covalent bonding. In pervaporation desalination, these crosslinked membranes presented significantly increased water flux as well as mechanical properties. However, the crosslinked membranes by hydrogen bonding did not prevent swelling because the nanochannel formed increased water uptake. Meanwhile, thermal crosslinking reduced the swelling ratio by 45–70%, while maintaining reasonable water flux and tensile strength. The S-SEBS with 15 wt% H302 treated at 100 °C reached a water flux of 9.34 kg m^−2^ h^−1^ and NaCl rejection of 99.9%.

Thi et al. [3] summarised sustainable membrane fabrication methods for organic solvent nanofiltration. Since polymeric membranes are the most widely used and fabricated, often by solution-based phase inversion techniques, the use of polar aprotic solvents such as DMF, NMP and DMAc has brought environmental concerns. Apart from developing membrane materials for greater performance, greener fabrication has been considered to improve the sustainability in membrane separation technology. Organic solvent nanofiltration has been regarded as an environmentally friendly separation process and an alternative to thermal distillation processes, but the requirement for membrane materials can be selective to deal with harsh chemicals for nanofiltration. The authors compared recent and traditional membrane fabrication methods and also suggested how to minimise environmental impacts during the fabrication process. The traditional and conventional polymers from petro-derived products can be substituted with bio-based polymers, such as collagen, chitosan, poly(lactic acid) and alginate polymers. Moreover, the use of greener solvents such as Rhodiasolv^®^ Polarclean, gamma-Valerolactone, methyl lactate, ethyl lactate, triethyl-phosphate, Cyrene, or ionic liquids can replace polar aprotic solvents for membrane fabrication. These alternative solvents are especially promising since some multinational pharmaceutical companies such as GSK have already reported the restriction in the use of toxic organic solvent in their manufacturing processes. Sustainable fabrication can be applied not only for the membranes for organic solvent nanofiltration, but also for the membranes for other applications such as ultrafiltration, desalination, or gas separation, such that the study in this area will become much more important in the near future.

Chuah et al. [4] reviewed graphene-based membranes for hydrogen separation. Hydrogen is an important industrial gas that has attracted attention as a carbon-free energy resource with the highest energy density per unit mass. Unlike conventional fossil fuels, hydrogen does not emit greenhouse gases after combustion. As most hydrogen production processes generate undesirable impurities such as carbon dioxide, methane, or nitrogen, the separation process is essential to produce pure hydrogen. Membrane processes have been proposed as alternatives to traditional pressure-swing adsorption and cryogenic distillation, but the performance of polymeric membranes has been limited in the production of high-purity hydrogen. In recent years, advanced membrane materials such as frameworks or two-dimensional materials have demonstrated great hydrogen separation performance. In particular, graphene-based membranes have presented outstanding hydrogen separation properties through the interlayer channels in graphene laminates. These membranes can be fabricated by spin-coating or vacuum filtration of water-based solutions on flat sheet substrates. For large-scale applications, hollow fibre types of substrates are also applied for vacuum filtration fabrication. There have been unresolved issues with large-scale membrane fabrication and applications of graphene-based membranes, and a lack of systematic analysis for realistic operation conditions, including the presence of water vapor or reactive components in the feed stream. Nevertheless, the potential of graphene-based membranes for practical industrial operations in hydrogen separation is promising.

Lastly, Volpin et al. [5] reported urine treatment applications on the International Space Station (ISS). They focused on membrane-based approaches with current challenges and opportunities. Due to limited resources and the enormous cost of shipment to space, recycling water and waste on the ISS is essential to reduce the value of payload mass. In this scenario, urine recovery and reuse can produce water for drinking and hygiene and generate on-site oxygen and nutrients for plant growing media as a safe fertiliser. Currently, the water recovery and management system reclaims wastewater, including urine, by distillation and filtration. However, this system may not be ideal because of the limitations in water recovery, resupply cost and maintenance. This review introduces and suggests current and advance urine and wastewater treatment systems on the ISS, including membrane-based systems by forward osmosis, reverse osmosis, osmotic distillation and membrane distillation. Recently-developed novel membranes such as aquaporin and graphene oxide are also capable of transforming wastes into usable and valuable products for plant production or radiation protection.

In conclusion, the findings and critical discussions from these contributions highlight the importance of membrane materials and the processes for water, gas and ion separation. Numerous materials and processes have already demonstrated their performance in various novel separation applications, and recent findings are under investigation for the in-depth characterisation and upscaling for practical applications. This Special Issue introduces guidelines for the sustainable development of these separation processes.
